# Contribution of terrestrial processes in reducing environmental mycotoxin levels: a review on mycotoxin-soil interactions

**DOI:** 10.1007/s12550-026-00635-3

**Published:** 2026-02-10

**Authors:** Katherine Muñoz, Sven Korz, Maximilian Meyer, Beatrice Berger

**Affiliations:** 1 Institute for Environmental Sciences (iES Landau), Department of Natural and Environmental Sciences, RPTU University of Kaiserslautern-Landau, Landau, 76829 Germany; 2 Institute for Plant Protection in Field Crops and Grassland, Federal Research Centre for Cultivated Plants, Julius Kühn-Institute (JKI), 38104 Braunschweig, Germany

**Keywords:** Mycotoxins, Soil, Biogeochemical processes, Secondary metabolites

## Abstract

Mycotoxins are fungal metabolites that can harm humans and animals. They occur primarily in infected crops, with higher levels under environmental stress. Their origin in contaminated crops is the main contributor to soil contamination. Although high levels can be detected in soils, these are transitory and in average far lower than in contaminated crops, indicating a key role of functioning soils in down-regulating environmental mycotoxin levels. This review examines mycotoxin-soil interactions in terms of four terrestrial processes: (i) adsorption to soil fractions, (ii) microbial transformation and mineralisation (iii) transport/mobilization with water, (iv) plant uptake and response. Results show that the main process for dissipation in soils is microbial, improved by biomass and activity and by a narrow fungi-to-bacteria ratio. This aligns with incubation studies suggesting a key role of bacteria in the degradation of mycotoxins. This path is favoured by the availability of the mycotoxins, that can be reduced by the adsorption to soil particles: the aromatic character and the carbonyl functions are decisive for adsorption to soil organic matter and clay particles, respectively. Consequently, mycotoxin levels in environmental compartments can be interpreted as the (net) result of entry and dissipation processes mediated through the soil, rather than the soil acting as a source of them. Plant uptake represents a minor pathway of contamination compared to the *in situ* production. In soil-plant systems, suboptimal nitrogen (N) and pesticide management are factors triggering fungal infestation and mycotoxins levels. Fungal infestation and mycotoxin can induce the polyphenolic response of the plant, along with mycotoxin metabolization as reported for DON. Based on this newly-made interpretation, this review provides future perspectives on mycotoxin occurrence and fate under climate change and intensive agriculture and proposes soil-based strategies for an integral environmental monitoring.

**Graphical Abstract:**

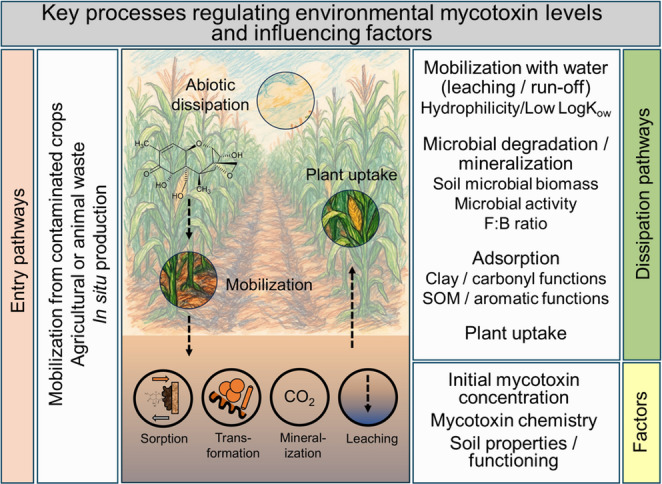

## Introduction

Mycotoxins form a heterogeneous group of secondary metabolites of fungal origin that can be toxic to humans and animals (Bennett and Klich 2003; Degen 2017). Species of the genera *Fusarium*,* Aspergillus* and *Penicillium* infest raw materials and food/feed commodities and are considered as the main producers of mycotoxins (Bräse et al. 2009). It is estimated that far more than 25% of the global food crop production is contaminated with mycotoxins (Eskola et al. 2020). Levels of mycotoxins in crops follow a geographic distribution (Raj et al. 2022), as fungal ecology and mycotoxin occurrence and diversity is influenced by climatic factors (Perrone et al. 2020). In tropical and subtropical conditions regions i.e. Sub-Saharan Africa, mycotoxin detection frequencies reach up to 40% in maize samples and with AFB1 (aflatoxin B1) levels in a range between 100 and 1000 µg kg^− 1^ and beyond (Omara et al. 2021), exceeding by 50–500% the maximum levels adopted in 2006 by the EU (Van Egmond et al. 2007). In temperate climates like in Europe, *Fusarium* diseases and its mycotoxins are in the focus with heterogeneous contamination levels: in Poland for example, deoxynivalenol (DON) and zearalenone (ZEN) were the most common mycotoxins detected in maize samples (97.3% and 98.4% detection frequency), with DON yearly mean levels between 21 and 45 µg kg^− 1^ (Twarużek et al. 2021), below the EU maximum limit of 1,000 µg kg^− 1^. This contrasts with the results reported by Hoppe et al. (2024), with DON values in maize grains in Poland in the range of mg kg^− 1^. Mycotoxin levels, independently of the geographical region, might worsen under the impact of climate change (Wu and Mitchell 2016), reaching levels of concern also in Europe e.g. *Fusarium* mycotoxins and aflatoxins (Focker et al. 2023). The predicted disease pressure and mycotoxin occurrence in crops in Europe may result in more serious health and economic issues (Johns et al. 2022). Consequently, research on mycotoxins focused on their occurrence in food and feed, the associated exposure of humans and animals, and elucidation of toxic effects and mechanisms of action. Different aspects of the plant-macro- and microorganisms interactions, including mycotoxigenic fungi, have also been described before e.g. by Sweany et al. (2022), Pfliegler et al. (2020). However, the soil ecosystem, has yet not been systematically studied, although mycotoxigenic fungi are an integral part of the soil microbiome. In addition, mycotoxins are part of the chemical ecological response of fungi in terrestrial ecosystems and evidence exists on the occurrence of mycotoxins in soils and in adjacent ecosystems.

Levels of mycotoxins in agricultural soils are 100–1000 times lower (< 30 µg kg^− 1^) (Juraschek et al. 2022) compared to levels in commercialized food and feed items. Luo et al. (2021) summarized data for DON reported in different European countries with average levels of 1,565 µg kg^−1^ in maize from Croatia, 2,687 µg kg^− 1^ for oats from Finland, 3,063 µg kg^− 1^ in maize samples from Serbia or 2,054.4 µg kg^− 1^ in maize silage from Spain. Levels reported for contaminated crops are e.g. 85 − 26,585 µg kg^− 1^ DON in wheat crops from Italy (Blandino et al. 2010), 4,860 µg kg^− 1^ fumonisin B1 (FB1) in 2018 and 1,453 µg kg^− 1^ in 2019 in freshly harvested maize grain from Uruguay (Del Palacio et al. 2023). Mobilization of mycotoxins from contaminated field crops to soil via leaching, run-off and wash-off, rainfall, irrigation or flooding remain the pathways with the highest contribution to soil contamination with mycotoxins (Kolpin et al. 2014; Schenzel et al. 2012a, c). Additional sources of contamination are the application of biosolids such as sewage sludge (Martín-Pozo et al. 2019), and animal manure (Kang’Ethe et al. 2017) and contaminated seed material. The use of food and agricultural waste as soil amendments in the context of circular economy (Palansooriya et al. 2023) can be also considered as a source for mycotoxin contamination of soils. The incorporation or ploughing of contaminated plant residues into the soil serves as a carrier for mycotoxins and fungi (Accinelli et al. 2008; Winter and Pereg 2019): Zhou et al. (2025) reported levels for mycotoxins in corn stover in the range of mg kg^− 1^ for ZEN and FB1. No-till soil management can result in an unintended risk for mycotoxin contamination as crop residues are not removed from the soil (Scott and Wu 2024).

Once in the soil, mycotoxins interact with abiotic and biotic soil components, which determines the residual and available levels. Mycotoxin adsorption to soil organic matter (SOM) (Schenzel et al. 2012b) and degradation by soil microorganisms (Ikunaga et al. 2011; Shima et al. 1997) are relevant environmental dissipation pathways. In addition, mycotoxins can be mobilized towards adjacent systems such as plants via plant uptake (Mantle 2000) or to streams via transport with water (Bucheli et al. 2008; Schenzel et al. 2012c). As these processes are not continuous, mycotoxins may be present in soils transiently at different levels of contamination and heterogeneously distributed (Kenngott et al. 2022). Dissipation processes through the soil account for low levels of mycotoxins and its metabolites in streams, in the range of ng L^− 1^ (Bucheli et al. 2008). Until now, the frequently reported mycotoxins in streams are DON (Schenzel et al. 2012a), ZEN (Gromadzka et al. 2009; Maragos 2012) and aflatoxins (Picardo et al. 2020). Other mycotoxins have not been detected or not included in the spectrum of analytes. This shows the need for further investigations to estimate the contribution of the land-water pathway in an indirect mycotoxin contamination.

From the perspective of fungal ecology, mycotoxins are secondary metabolites synthesized by the fungus to compensate biotic and abiotic stresses in terrestrial ecosystems (Venkatesh and Keller 2019). *Fusarium*,* Aspergillus and Penicillium* are an integral part of soil microbiota (Christensen et al. 2000; Klich 2002; LeBlanc et al. 2017) and known to produce a wide spectrum of mycotoxins when colonizing environmental matrices (Bräse et al. 2009). But an *in situ* production of mycotoxins in soil has not been documented. Mycotoxins are known to increase the virulence of fungi (López-Berges et al. 2013) to provide a competitive advantage over other organisms as a chemical shield (Boellmann et al. 2010; Trienens et al. 2010) or to enhance the survivability of spores under environmental stress conditions (Calvo et al. 2002). They can act as allelochemicals, compounds released by a living organism in the environment, i.e. fungi, affecting plants and other (micro)organisms (Yoneyama and Natsume 2013). This property was described by Polyak and Sukcharevich (2019) for rubratoxin, sterigmatocystin, patulin (PAT), citrinin (CIT), ochratoxin A (OTA), among other secondary metabolites. Furthermore, toxicity of mycotoxins to soil biota at reported environmental levels was absent (Abid et al. 2021; Albert et al. 2023; Meyer-Wolfarth et al. 2021).

Mycotoxin production by filamentous fungi is shaped by abiotic factors e.g. high temperatures (Selouane et al. 2009), osmolarity differences (Stoll et al. 2013), high CO_2_ concentrations (Magan and Medina 2016), unusual light fluxes (Fanelli et al. 2012), or the use of fungicides (Schmidt-Heydt et al. 2013; Simpson et al. 2001). These parameters are frequently modified in modern agriculture toward better crop productivity with consequent shifting of soil processes. Accordingly, intensive agricultural practices and climate change are described as major drivers of soil disservices. In this context, there is unanimity that environmental factors such as increasing temperatures, stress scenarios of drought (Kolawole et al. 2021) and flooding as well as CO_2_ atmospheric concentrations (Medina et al. 2015b) will go along with a higher fungal infestation and mycotoxin production in crops. Regardless of the scale or the factors studied, the majority of authors agree that mycotoxin levels in crops will increase as a result of changing climatic stress conditions (Casu et al. 2024; Medina et al. 2015a; Moretti et al. 2019; Paterson and Lima 2010). Thus, high levels of mycotoxins in crops can be a starting point for high levels of mycotoxins in different environmental compartments, i.e. soil and streams.

The aim of this review is to explore the soil-mycotoxin interactions, extending beyond the sources of contamination or reported environmental levels. Specifically, it focuses on the potential of soil to reduce environmental mycotoxin levels, rather than acting as a source of mycotoxins. Therefore, the role of soil components, both biotic and abiotic, in regulating environmental mycotoxin levels is addressed, considering processes such as mobility and transport, adsorption, (bio) degradation and metabolism within the soil to the plant, and within the plant. In addition, factors that influence the mycotoxin production by fungi in soils and plants, with focus on the ecological role of mycotoxins for *Fusarium* are described. How plants respond to fungi/mycotoxins with the formation of plant secondary metabolites is addressed to complement the ecological role of mycotoxins in terrestrial ecosystems. Finally, we elaborate on future considerations for sampling strategies and analysis of mycotoxins in environmental samples, including the role of climate change and intensive agriculture in environmental concentrations of mycotoxins.

## Terrestrial processes shaping environmental levels of mycotoxins

Mycotoxin-soil interactions involve a variety of processes that determine the levels of mycotoxins in different environmental compartments. One important process is dissipation, which greatly contributes to reducing the initial mycotoxin concentrations, either temporarily or permanently. This section describes the adsorption and microbiological degradation as the main pathways by which mycotoxins are dissipated from soils. Additional dissipation pathways include those mediated by the transport with water to adjacent ecosystems namely streams and by plant uptake.

### Sorption of mycotoxins to soil particles and fractions

Soil contains different constituents that can act as sorbents: natural polymers such as chitosan and cellulose (Solís-Cruz et al. 2017), clay or clay mixtures (Holanda and Kim 2020; Santos et al. 2011). Humic substances belong to the SOM and consist of complex macromolecules with strongly varying composition, that usually contain an aliphatic backbone to which various organic functional groups are bound, for example, COOH, -CO, CHO, or OH, along with aromatic groups (Aquino et al. 2011). Clay minerals are also a relevant constituent of the soil that interact with organic and inorganic compounds by adsorption, intercalation and cation exchange (Lagaly 1984).

Mycotoxins adsorb to soils, but its sorption potential is linked to the physicochemical properties of the mycotoxin. In a column experiment with peat as model for organic matter, mycotoxins with a Log*K*_oc_ between < 0.7 to 3.5 were investigated, with DON showing the lowest retention to peat and verrucarin A the highest (Schenzel et al. 2012b). The authors indicate that structural differences between the mycotoxins influenced the Log*K*_oc_ values, with -OH groups, i.e. DON, lowering the value and increasing H-bridge interactions with water molecules. The Log*K*_oc_ -partition coefficient normalized to organic carbon- is a suitable predictor of mycotoxin sorption to soil when studying the fate of mycotoxins (Schenzel et al. 2012b), but this reasoning is not applicable to all mycotoxins. The mycotoxin DON (Log*K*_ow_ −0.93) was almost not adsorbed to soil, meanwhile 15-ADON (Log*K*_ow_ −0.63) showed sorption patterns from 21 to 97% increasing in function of clay content in the soils (Korz and Muñoz 2025). By contrary, the adsorption of ZEN (Log*K*_ow_ 3.83) was above 90% and was not affected by the soil type, indicating two different types of non-covalent interactions: (i) cross-linking of organo-mineral associations (Kunhi Mouvenchery et al. 2012) between the carbonyl group in 15-ADON and cations present with increasing clay content. (ii) molecules with aromatic similarities as humic acids like AFs, CIT, OTA, ZEN, sterigmatocystin and *Alternaria* toxins have the additional capacity to adsorb to organic matter through interactions of aromatic π-donor and -acceptor (Keiluweit and Kleber 2009), resulting in an increased sorption potential in function with the organic matter content in the soil (Keiluweit and Kleber 2009).

The Log*K*_ow_ along, cannot predict the fate in soils: the mycotoxin AFB1 (Log*K*_ow_ 1.23) was detected in water samples from columns containing 10 and 20% silty clay loam soil. But, FB1 (Log*K*_ow_ −0.77), could not be quantified in those samples despite its higher water solubility compared to AFB1 (Madden and Stahr 1993). The chemical similarity between the functional groups in FB1 and humic substances contributes to better retention in soil (Deshmukh et al. 2007). This aligns with the finding of Williams et al. (2003), showing a tight binding of FB1 (100% retention) to cecil sandy loam in a soil column leaching experiment. However, the binding was reversible under acidic pH value conditions like those for formic acid, suggesting non-covalent and a pH-dependent interactions between soil fractions and mycotoxins. Cross-linking of organo-mineral associations has been suggested to explain nonlinear sorption of organic chemicals with carbonyl functions to soils (Kunhi Mouvenchery et al. 2012). Soils are characterized by a wide range of pH (< 3.0 - ~11) (Kuntze et al. 1994), which is relevant when assessing the fate of molecules with ionizable groups (Franco et al. 2009), like FB1 and its carboxylic functions. The deprotonation potential of mycotoxins in this pH range can be derived from the p*K*a values (Table 1). For example, when the pH value is higher than the p*K*a the functional group undergoes deprotonation, favouring ionic interactions. Also in streams, different pH values can influence the abiotic and biotic degradation kinetics of ionizable molecules (Baena-Nogueras et al. 2017).

**Table 1:**
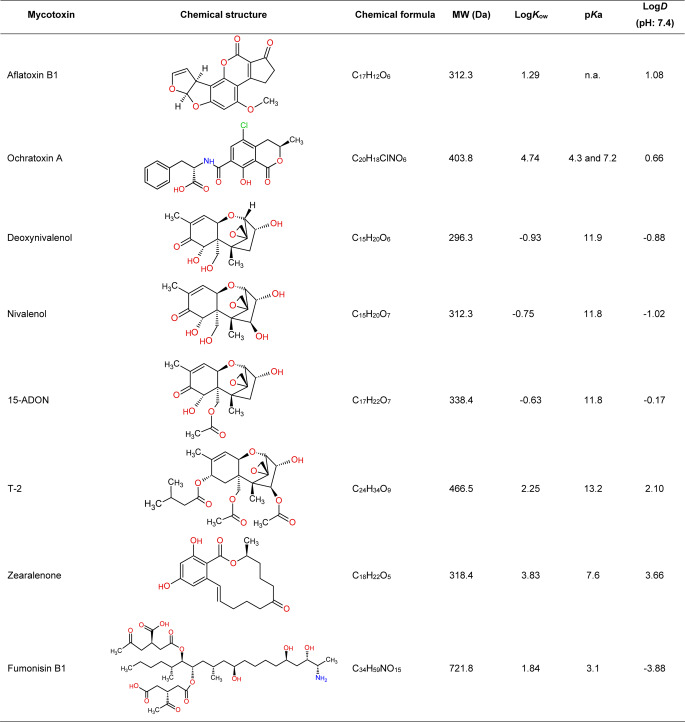
Physical and chemical properties for selected mycotoxins. Structures were provided by ChemSketch, and values were obtained using the predictors ACD/Labs

Mycotoxins have mostly a Log*K*_ow_ < 5 grouping them the range of non-persistent organic pollutants (Baker et al. 2000). Thus, we can expect that the structure and properties of the soil will play a crucial role in determining the availability of the mycotoxin and the extent of additional processes. In brief, this research highlighted the role of soil texture i.e. (i) clay context in the interaction with mycotoxins owning carbonyl functional groups in its structure. And (ii) the chemical properties of the soil such as organic matter content on the fate of mycotoxins particularly those with aromatic functions. This work also indicates that empirical data from different soils is needed to precise on their fate, and that Log*K*_ow_ data alone might fail in predicting adsorption and desorption processes e.g. in clay soils (Korz and Muñoz 2025). For ionisable molecules (OTA, ZEN, FBs), which are likely to be charged in the environmental pH value range, Log *D* values may be more accurate as the partitioning becomes pH dependent (Xing and Glen 2002). This understanding is relevant when designing monitoring strategies to assess the occurrence and distribution of mycotoxins in the environmental compartments. In addition, there is so far no information how adsorption processes work in soils under different managements, for example using soil amendments which increase the organic carbon content in soil or those modifying hydraulic and climatic properties. It should be further considered that mycotoxins are bound to soil fractions via non-covalent interactions, and that tight interactions with soil fractions are reversible, with a considerable fraction of the mycotoxins available in the water phase for further processes such as (bio)degradation, transport and uptake.

### Microbial transformation and mineralization by microorganisms isolated from soils

**Fig. 1:**
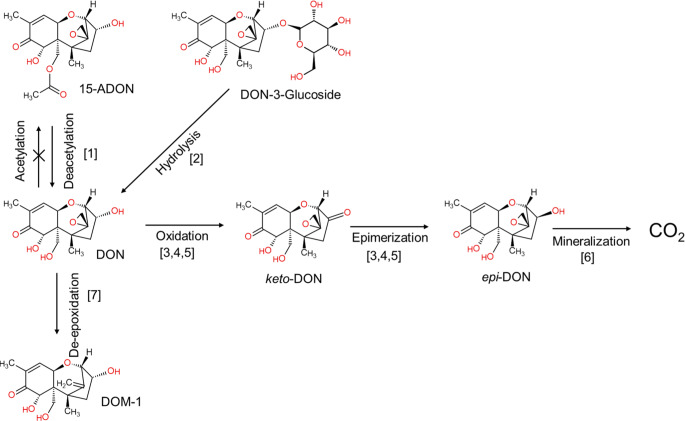
Putative pathways of DON microbial transformation by soil microorganisms. [1] Deacetylation described by Korz and Muñoz (2025) in three different agricultural soils, no reports exist currently describing an acetylation of mycotoxins. [2] The hydrolysis correspond to a possible pathway derived from evidence in plant secondary metabolites, such as flavonoids, with a cleavage half-life < 2 h (Kong et al. 2007). This pathway has not been demonstrated for mycotoxin glycosides. [3, 4] The conversion of DON to *epi*-DON via the intermediate *keto*-DON was described by Shima et al. (1997) and later by He et al. (2024a) both in culture experiments and by [5] Kenngott and Muñoz (2024) in maize cultivated soils. [6] Mineralization of DON, meaning the conversion of C source to CO_2_, was reported by different authors and summarized by Karlovsky (2011). [6] The epoxidation of DON is an anaerobic process, that can be possible in soil under limited O_2_ conditions. DOM-1 values in soils are linked to animal manure (Wang et al. 2022)

DON can be effectively degraded by microorganisms isolated from the soil. Shima et al. (1997) reported ~ 70% of conversion of DON to 3-keto-DON with the formation of *epi*-DON, the epimer of DON (Fig. 1), being the modified forms less toxic or nearly non toxic to other microorganisms when compared with the parent compound. The conversion was done by a Gram-negative E3-39 species belonging to the *Agrobacterium-Rhizobium* group cultured for two days in BYE agar containing 200 µg mL^− 1^ DON. In the same experiment, the trichothecene 3-ADON was also metabolized by E3-39 but at a lower rate than DON, whilst NIV and fusarenon-X were not metabolized. This indicated that small chemical modifications i.e. the additional OH-group in NIV compared to DON was decisive in the degradation potential by this soil microorganisms. Similar results for the microbial transformation of DON were reported for the bacterium *Devosia sp.* D6-9 (He et al. 2024b), for *Devosia insulae* FS10-7 under a wide range of pH (4 to 10) and temperature (16 to 42 °C) (He et al. 2024a). *Devosia spp.* are Gram-negative aerobic bacteria isolated from soils (Yoon et al. 2007). The bacterium WSN05-2, closely related to *Nocardioides*, isolated from an experimental wheat field soil showed a nearly fully efficacy to degrade DON (10–1000 µg mL^− 1^) in modified mineral medium (Ikunaga et al. 2011). The authors reported that the growth of WSN05-2 was directly proportional to the amount of DON, and that DON-metabolites were not detectable after 10 days, suggesting a utilization of the mycotoxin for energy in form of mineralization. A reduced transformation of DON in function of the concentrations was also reported by Wang et al. (2023a).

The mineralization of DON, transformation to CO_2_, by soil microorganisms was summarized by Karlovsky (2011), pointing out the need to differentiate between processes i.e. mineralization and adsorption, as well as assimilation and absorption. Sato et al. (2012) observed that Gram-positive strains, closely related to the genus *Nocardioides* in the family *Nocardioidaceae*, utilize DON in minimal medium (MM) as solely carbon source at a concentration of 100 µg mL^− 1^, contrary to the Gram-negative genus *Devosia*, favouring the transformation of DON in *epi*-DON over a mineralization. The strains used were collected from a wheat field soil, a paddy field soil, an uncultivated soil (at a shrine), and from wheat leaves and spikelets. Among the strains investigated, clear DON-degradation phenotypes within closely related strains were observed. Not all strains tested show this ability, suggesting selective substrate utilization by Gram-positive and Gram-negative strains (Kramer and Gleixner 2008). The utilization of DON as single C-source was also described by He et al. (2008) for the fungal specie *A. tubingensis* isolated from agricultural soils.

Another microbial transformation described for DON is the de-epoxidation, a reductive process that involves the conversion of the epoxide to a carbon–carbon double bound. The conversion of DON (64.3%) to DOM-1 was observed by Islam et al. (2012) at a 50 µg mL^− 1^ concentration after 72 h of incubation using a soil suspension in two incubation media (NB and MSB). De-epoxidation occurred in a wide range of temperature (12 to 40 °C), but in a narrow range of pH (6.0–7.5). DOM-1 is a frequently reported metabolite generated in the intestinal tract of ruminants and less common in humans and pigs (Wang et al. 2022), whose formation is mostly associated to anaerobic conditions. But, Islam et al. (2012) reported even a more efficient de-epoxidation under aerobic conditions by a bacterial consortium (*Serratia*, *Clostridium*, *Citrobacter*, *Enterococcus*, *Stenotrophomonas* and *Streptomyces*) in a composite sample from different agricultural soils. In soils, aerobic conditions govern the processes and favouring oxidative reactions. Therefore, DOM-1 levels in soils can be attributed mostly to a secondary origin like manure than being a consequence of *in situ* metabolism. The above *in vitro* results indicate the ability of soil bacteria for degrading DON and other trichothecenes.

Microbial transformation is also influenced by the composition of the microbial consortium. For example, a two-bacteria consortium model (*Pseudomonas* and *Devosia* from a faecal insect sample) was capable of degrading 86.6% DON with a maximum activity at 30 °C and within a pH range of 8 to 9 (Wang et al. 2023b), being more effective than the single strains. Furthermore, the degradation rate for DON was reported to be more efficient in the presence of mixed bacteria compared to a single bacterium (Wang et al. 2023a; Yao et al. 2024). Zhai et al. (2019) described a two-steps degradation of DON, indicative of a process with a cooperative mechanism between Y1 (*Pseudomonas sp*., 44.98%) and S1 (*Lysobacter sp*., 0.11%). Bumunang et al. (2024) reported that the capability of bacterial consortia for DON degradation is strongly linked to the soil source. The addition of available C-sources like alcohols and sugars reduced their degradation efficiency, suggesting the utilization of DON as C-source and thus a mineralization of the mycotoxin. The same group observed that a bacteria consortium consisting of *Devosia sp.* A8 and *Paracoccus yeei* A9 was able to degrade for 3-ADON (49.7%), 15-ADON (97.4%) and T-2 (22.4%), but not HT-2 degradation (Wang et al. 2023a), despite of close structural similarities of these mycotoxins (Table 1). This is in line with other studies showing a chemical selectivity in mycotoxin degradation. In the case of T-2 vs. HT-2 the differences in degradation can be attributed to a de-acylation (Table 1), the first detoxification steps for acetylated trichothecenes e.g. Vanhoutte et al. (2016). Deacetylation of the Toxin T-2 in HT-2 and later T-2 triol was described by Ueno et al. (1983). This implies the biotransformation potential is not restricted to single microbial species but to an interplay of microorganisms in a given consortium. Figure 1 summarizes putative microbial pathways of DON metabolism in soils.

Complete degradation of OTA into OTα was described for microbial populations from three vineyard soil samples, when grown in MM Peptone (De Bellis et al. 2015). Degradation of ZEN was achieved by *Pseudomonas alcaliphila* and *P. plecoglossicida* isolated from soils, with a 50% degradation after 72 h and similar degradation rate (Tan et al. 2014). Both strains shared oxidase, catalase and dihydrolase activities, suggesting a redox reaction for chemical conversion. *Klebsiella pneumoniae* isolated from agricultural soils was able to fully degrade ZEN, with greater efficiency at higher temperatures and pH values (Imade et al. 2023). Other strains with ZEN-degrading capacity are *Bacillus amyloliquefaciens* isolated from wheat soils (Xu et al. 2016), with a negative correlation in function of ZEN-concentration and suggesting a mineralization instead of a biotransformation. Most of the methods indicate the absence of the parent compound as a positive result of its degradation, without exploring potential metabolites. Yet, Vekiru et al. (2010) described a metabolite of ZEN, ZOM-1, with a more polar character than ZEN and a shorter retention time in reverse phase columns. The metabolite ZOM-1 is formed by a process involving an oxidation and a hydrolysis step (Table 2). *Bacillus subtilis* from agricultural soils was able to degrade AFB1 due to the extracellular enzymes without identification of breakdown products via LC-MS (Xia et al. 2017). Arimboor (2024) summarized in a review the major microbial pathways for AFB1 breakdown. The author described different enzymatic processes including oxidation, reduction, de-methylation, hydrolysis, etc. The breakdown of AFB1 involved a mass shift of up to 206 Da, with putative metabolites described as biotransformation products. Metabolites reported for selected mycotoxins, including detection methods are summarized in Table 2.

**Table 2:**
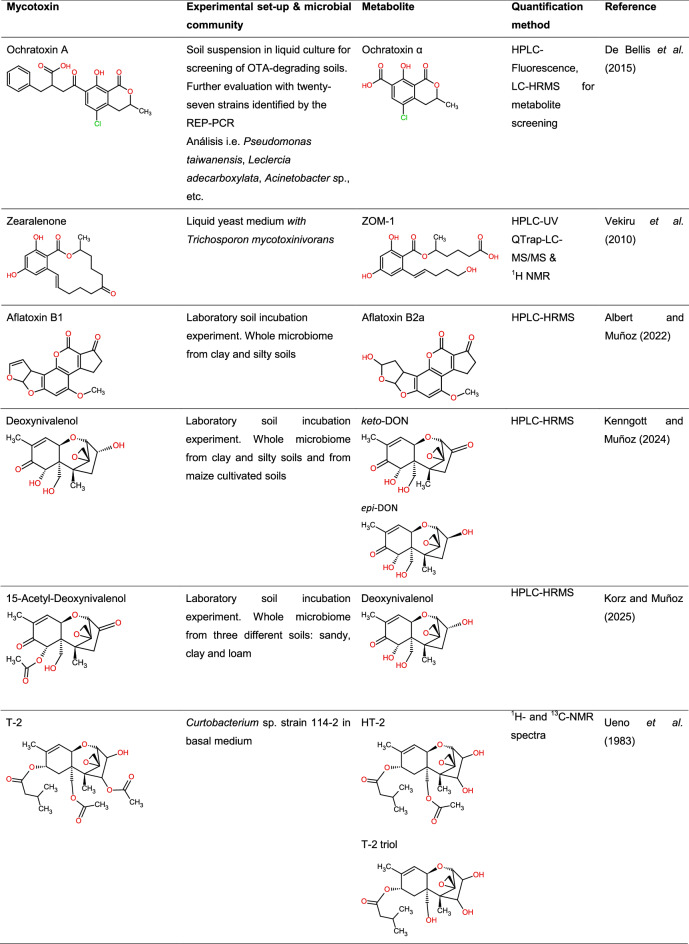
Degradation of selected mycotoxins as mediated by soil microorganisms including suggested metabolites and analytical methods

### Microbial degradation and mineralization in soils

Degradation rates in soils (days) are slower than those reported for culture experiments (hours), divergence that can be explained by adsorption processes to soil organic matter or clay fractions, restricting the availability of the mycotoxins for biogeochemical processes (Albert and Muñoz 2022). In the case of DON, there is almost no adsorption to soil particles (Schenzel et al. 2012b), suggesting a high (almost 100%) availability for microbial processes. Dissipation rates of 50% (DT_50_) were achieved for DON after 0.6–3.7 days in soils from maize fields (Kenngott and Muñoz 2024), while it took six months to reach 100% dissipation in a meadow soil (Abid et al. 2021). Differences are attributed to study set ups, *inter alia* microcosm size, soil type and management (arable land vs. meadow), conditions (50% vs. 80% water holding capacity), soil amendments (straw incorporation), etc. and are in line with the results of (Wang et al. 2023b), where the degradation of DON was restricted with increasing available C-sources such as trehalose, lactose, sucrose, maltose, mannitol, and glucose. DON, NIV and 15-ADON were effectively degraded in three soils with degradation aligned with the soil microbial biomass and respiration in that order sandy loam > silty loam > silty clay (Korz and Muñoz 2025). Independently of the soil type, 15-ADON was fast converted to DON reaching the lowest DT_50_ value (< 0.2 days), indicating that the de-acetylation is a generic transformation pathway, also applicable to mycotoxins with similar functionalities. Yet, all authors concluded that dissipation was primarily due to microorganisms, since no degradation nor absorption was observed in the sterile set-ups. The dissipation of DON in soils is consistent with the findings by Meyer-Wolfarth et al. (2021), who reported no recovery of DON in leachate or soil. The degradation of DON was enhanced in soils with active and large microbial communities and a low fungi-to-bacteria ratio (Kenngott and Muñoz 2024). Furthermore, the authors indicated that the degradation rates in soils from maize fields were more efficient than in the standard agricultural soils with no legacy of maize cropping, suggesting a priming effect in the former. In conclusion, the current evidence indicates that the microbial composition and the activity of the soil microbial community, influence the degradation of DON.

**Fig. 2:**
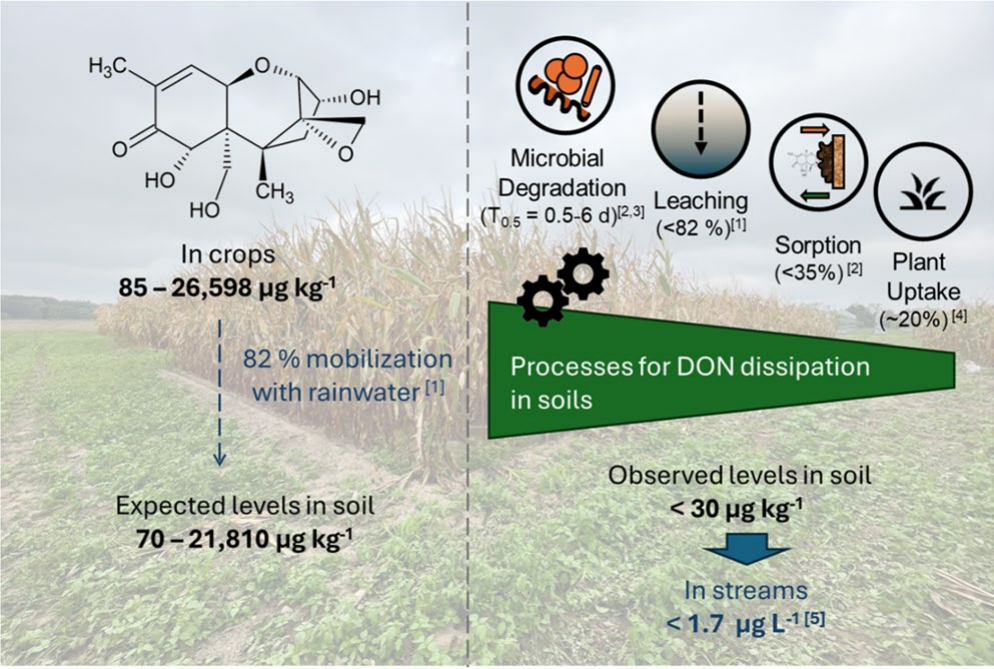
Processes for dissipation of DON in soils. DON levels in crops were obtained from the study from Blandino et al. (2010). The mobilization of DON with water as well as leaching rate (82%) [1] was calculated in a leaching experiment by Meyer-Wolfarth et al. (2021). Values for microbial degradation [2, 3] provided by Kenngott and Muñoz (2024) and Korz and Muñoz (2025). Data on plant uptake [4] obtained from Righetti et al. (2021a) and values for DON levels in streams [5] as reported by Kolpin et al. (2010)

Agricultural managements such as straw incorporation enhanced DON degradation in soil (Abid et al. 2021). Since straw provides C and N inputs, which might increase microbial biomass and activity (Muñoz et al. 2022), a higher dissipation rate can be expected. This aligns with a faster DON dissipation observed for straw-covered than plastic-covered soils in strawberry cultivation (Meyer et al. 2021). This may pose a contradiction to that reported by (Wang et al. 2023b), but in this case straw corresponds to a complex C-input other than simple C-compounds like sugars or amino acids, so that a decomposition is needed, allowing simultaneous microbial processes such as assimilation and biomass increase, along with a higher respiration rates (Korz et al. 2024). Figure 2 shows the extent of DON dissipation by four soil processes (microbial degradation, transport mobility with water, sorption and plant uptake. The figure describes a scenario of DON contamination in wheat crops, with an initial concentration up to 26,598 µg kg^− 1^ (Blandino et al. 2010). DON is highly mobilized with water with a leaching potential through the soil column up 82% (Meyer-Wolfarth et al. 2021). Considering those factors, levels in soils should be in the range of ~ up to 21,810 µg kg^− 1^. But, concentrations in soils from agricultural fields indicate levels 100–1000 times lower (Kenngott et al. 2022). This example indicates that only a minor fraction of the initial mycotoxin concentrations in crops reach adjacent streams, suggesting a pivotal role of soil in reducing levels, mainly due to microbial degradation.

Degradation in soil was also confirmed for other groups of mycotoxins. Accinelli et al. (2008) found AFB1 levels in agricultural soil ranging from 0.6 to 5.5 ng g^− 1^ dry weight, with 60% of *A. flavus* isolates being aflatoxigenic. In their laboratory experiments, AFB1 had a half-life of five days in non-autoclaved silt loam soil at 28 °C. In a soil incubation experiment by Angle and Wagner (1980), AFB1 was degraded to a metabolite assigned by the authors via TLC analysis as AFB2, becoming undetectable after 77 days, with a fraction of 14% AFB1 being mineralized without added C-sources. Unlike DON, AFB1 dissipation was unaffected by the addition of straw or glucose. AFB1 mineralization depended on soil type, with the lowest mineralization observed for silty clay loam (Angle 1986). The reason for that might be the adsorption of AFB1 to clay particles, reducing its availability for microbial processes. AFB1 degradation in clay soil was less efficient than in sandy loam (Albert and Muñoz 2022). Nevertheless, the transformation product AFB2a was detected, and it was assumed that other metabolites may occur since the AFB2a concentration calculated accounted only for 20% of the parent compound. However, it remained unclear which portions of the overall decay were due to biotransformation, mineralization, biomass incorporation or formation of non-extractable residues as in soil aging processes. The utilization of AFB1 as a source of carbon and energy by bacterial isolates was early reported (Line and Brackett 1995), indicating the potential for mineralization.

It is crucial to elucidate factors shaping mycotoxin degradation, as mycotoxins occur in terrestrial and aquatic systems. Although a strong accumulation of mycotoxins across soils seems unlikely (Log*K*_ow_ < 5), single hot-spots with high mycotoxin concentrations may pose a risk for some microorganisms (Venkatesh and Keller 2019). These hotspots can occur in agricultural settings in regions with high mycotoxin levels, as contaminated residues can be incorporated into the soil. The degradation of mycotoxins is mainly driven by the soil microbiome, suggesting that soil functionality is key to regulating environmental levels of mycotoxins. This should be taken into account as intensive agricultural practices may interfere with processes in soils, reducing the potential for soil functioning (Yang et al. 2020).

### Mobilization and transport with water and levels in streams

Mycotoxin levels in streams (Table 3) are comparable to levels of fungicides mobilized from crops into streams (Battaglin et al. 2011; Lefrancq et al. 2014). Polar mycotoxins are more likely to migrate from fields through the soil column than non-polar mycotoxins (Schenzel et al. 2012b), based on the principle *“Like dissolves like”*. DON is chemically a polar molecule due to several OH-groups and frequently detected in streams (Table 3). Its levels in drainage water samples from contaminated fields range from 0.8 to 1,140 ng L^− 1^ (Schenzel et al. 2012a), which is less than 0.5% of the initial concentration in contaminated crops. Bucheli et al. (2008) showed a concentration for DON in drainage water from an inoculated winter wheat field ranging from 23 ng L^− 1^ to 4.9 µg L^− 1^ being up to 22 ng L^− 1^ in streams, accounting for a significant reduction in the land to water pathway. For ZEN, Hartmann et al. (2008) estimated fractions between 0.001 and 0.07% in drainage water, with concentrations up to 35 ng L^− 1^. Waśkiewicz et al. (2015) reported concentrations up to 48.2 ng L^− 1^ for FB1 in drainage water from wheat fields, whereas FB2 and FB3 could not be detected. In a field experiment, DON and 15-ADON (up to 3,500 ng L^− 1^ for DON) were detected in the run-off water after rain-fall (Gautam and Dill-Macky 2012), reflecting an small fraction of the mycotoxin levels in the fields. Mobilization and transport with water was also visible after harvest with ZEN originated from maize crop residues (Hartmann et al. 2008). The authors of this study also mention the soil bound fraction of ZEN being unavailable to aqueous elution.

**Table 3:** Mycotoxin concentrations reported for water samples

Source	Mycotoxin	Concentration (ng L^− 1^)	Reference
Swiss surface waters	Deoxynivalenol	n.d. – 22	Bucheli et al. (2008)
	Zearalenone	> LOQ	
River estuary, Portugal	Deoxynivalenol Zearalenone α-Zearalenol	59.5–373.5 < 137.5 < 96.3	Ribeiro et al. (2016)
Streams across Iowa, USA	Deoxynivalenol α-Zearalenol β-Zearalenol	n.d. – 1,662 n.d. – 1,701 n.d. – 1,828	Kolpin et al. (2014)
	Zearalenone	n.d. – 43.7	Gromadzka et al. (2009)
Ditches, drainage wells or lakes and rivers located in agricultural areas, Wielkopolska region, Poland	Fumonisins B1	n.d. – 48.2	Waśkiewicz et al. (2015)
Freshwater ponds (Flanders, Belgium)	Enniatin A1 Enniatin B Enniatin B1	n.d. – 2.6 n.d. – 6.9 n.d. – 3.3	Goessens et al. (2021)
Rivers and creeks (Portugal)	Zearalenone	5.6–82.6	Laranjeiro et al. (2018)

At the laboratory scale, mobilization of mycotoxins in water through leaching via the soil column has been described. In a Haplic Luvisol soil column (pH 7.9, texture 19% clay, 68% silt and 13% sand), over 80% of DON was leached from the contaminated wheat material after the simulation of a rain-event (Meyer-Wolfarth et al. 2021), whereas for ZEN no leaching could be demonstrated. The DON and ZEN values observed in the soils were not indicative of sorption, which would indicate additional processes, for example degradation or strong binding of ZEN to soil. Williams et al. (2003) detected higher leaching rates of FBs in sandy soils and at low pH values compared to other soils, suggesting a direct relation between leaching rates and soil properties.

Regardless of the chemistry and properties of the mycotoxin, i.e. DON vs. ZEN, the fraction of mycotoxin discharged with the water represents less than 1% of levels in the crops (Schenzel et al. 2012a). Yet, the occurrence of mycotoxins in effluent and drainage water is investigated by analysing aglycones, although active glycosylation and acetylation modifications *in planta* have been described for certain mycotoxins, i.e. ZEN, DON (Zhang et al. 2020). This may lead to an underestimation of levels coming from contaminated fields. Furthermore, the stability of mycotoxins as aglycones and conjugates in the water has not been investigated. Finally, passage through soil and the processes adsorption to SOM or soil particles and microbial degradation are effective to reduce mycotoxin concentrations reaching streams and merits further investigation.

### Translocation from soil to plant and biotransformation *in planta*

Mycotoxins that are not immobilized via sorption processes are available for biological interactions. There are several reports showing the uptake of mycotoxins by various plants and their transport to aerial parts. OTA was taken up by the roots in coffee plants in a soil experiment (Mantle 2000). FB1 was observed in symptom-free asparagus plants, which may suggest mycotoxin translocation from soil to plant (Waskiewicz et al. 2013). The mycotoxin was also reported in maize seedlings when FB1 was administered in the watering solution (Zitomer et al. 2010). The uptake and translocation of citric acid, patulin and terpenoids to shoots was observed when roots were dipped in test tubes containing the mycotoxins (Rao et al. 1982). Approximately 24 ~ 66% of the AFs added to the incubation medium was taken up by the excised roots after 18 h from the mycotoxin containing medium (Walker et al. 1984), an uptake rate similar to this was found in sugar cane under greenhouse conditions (Hariprasad et al. 2015). An *in vitro* experiment with xylem demonstrated uptake of AFB1 by groundnut plant roots and its translocation to the shoots (Snigdha et al. 2015). In plant incubation experiments using hydroponic systems, Righetti et al. (2017) described an effective absorption of ZEN in wheat leaves and roots fractions immersed in a mycotoxin solution of 12.5 µg L^− 1^ and 100 µg L^− 1^. The authors reported later that ZEN was almost completely absorbed after 14 days by wheat plants, while DON remained unaltered in the hydroponic solution (Righetti et al. 2021a). In the same set-up AFB1 was found to be almost completely absorbed after 14 days, with only 2% of the mycotoxin left in the solution (Righetti et al. 2021b). After 12 h incubation, approximately 60% of the supplied ZEN (Log*K*_ow_ = 3.83) was absorbed from the hydroponic solution, irrespective of the initial mycotoxin concentration (Rolli et al., 2018). T-2 (Log*K*_ow_ = 2.25) and HT-2 (Log*K*_ow_ = 2.27) absorption capability was investigated in plant organs of durum wheat (Righetti et al. 2019): T-2 in solution appeared to be taken up rapidly by the roots, whereas less efficient uptake was observed in the leaves. On the other hand, removal of HT-2 from the medium was slower and less efficient in both organs. Similar to ZEN, alternariol monomethyl ether (AME, Log*K*_ow_ 3.68), accumulated in the wheat roots and was subsequently transported throughout the plant up to the leaves after one week incubation in an hydroponic system (Jaster-Keller et al. 2023). Righetti and colleagues (2019) discussed also the differences in uptake for T-2 and HT-2, based on a maximum translocation for neutral chemicals with a Log*K*_ow_ of ∼1.78 as proposed by Briggs et al. (1982) for barley.

The plant fractions are interpreted as “biofactories”, as a wide range of metabolites can be detected, including those from redox, dehydrogenation and conjugation reactions (Righetti et al. 2020). Independent of the system used, the capacity of the plant or plant organs to biotransform mycotoxins has been demonstrated e.g. Li et al. (2020). The mycotoxins T-2 and HT-2 were biotransformed *in planta* into a total number of respectively 26 and 23 metabolites plus tentative isomers (Righetti et al. 2019). The authors used UHPLC-HRMS in full scan mass spectrum, further analysis for structure elucidation were not conducted. Similarly, Rolli et al. (2018) investigated the biotransformation of ZEN *in planta*, using non-target analysis with LC-HRMS full scan spectra: 7 putative phase I and 18 phase II metabolites were reported. The authors of this study suggested that biotransformation may serve to facilitate excretion for unwanted compounds, being part of the defence strategy of the plant. A high potential of biotransformation, including functionalization with incorporation of OH-groups or defunctionalization via hydrogenation, has been reported for ZEN (Righetti et al. 2017) and AFB1, with the formation of hydroxylated forms (Righetti et al. 2021b).

O-glycosylation has been reported as an effective biotransformation of mycotoxins *in planta* and described as “modified mycotoxins”. The formation of glucosides is a common conjugation reaction for mycotoxins with a free OH-group in its structure, for that reason, this type transformation is restricted to certain mycotoxins: *Fusarium* toxins i.e. DON, NIV, ZEN; *Alternaria* toxins, namely alternariol (AOH), AME and tenuazonic acid (TA) and OTA. The active metabolism described *in planta* justifies the exploration of modified forms in food commodities (Berthiller et al. 2013; Tan et al. 2023), but also their occurrence, stability and effects in the environment. Mycotoxins are actively metabolized in the host plant, reducing the concentration of the parent compound but generating diverse metabolites which are more polar than the parent compound.

Most of the studies investigating translocation from soil to plant use model systems without soils, although soil and its processes actively contribute to interactions between soil and mycotoxins. For a better comparability of data, factors such as plant species, biotransformation *in planta*, also as influenced by stress conditions, and chemical stability of mycotoxins should be evaluated in uptake experiments (Bagheri et al. 2021). The plant as influencing factor should also be evaluated in its function as host for the pathogenic fungi, considering ecological defence mechanisms that may affect fungal biomass/distribution along with the production of mycotoxins.

## Environmental factors triggering mycotoxin production and the role of plant defence

Chemical factors such as nutrient type and level or the simultaneous occurrence of chemical stressors can trigger the production of mycotoxins. Known is the reported effect of N in the virulence of *Fusarium in planta* and in the mycotoxin levels in crops. The use of certain fungicides and the dose and timing of application have been optimized in the last years aiming to reduce the levels of mycotoxins. Pathogenic fungi respond to this chemical stress with biosynthesis of secondary metabolites (Audenaert et al. 2014). This response, so far observed in incubation experiments and in the plant can be extrapolated to the soil. Soils are frequently contaminated with fungicides and mycotoxigenic fungi are part of the soil microbiome. Mycotoxins can be considered as chemical stressors triggering a response in the plant. Understanding how mycotoxins alter plant defence reactions, and how plant-derived compounds influence mycotoxin production is important for developing antifungal strategies. Among the plant defence mechanisms, secondary metabolites, particularly phenolic acids, have been identified as potential inhibitors of mycotoxin production. Phenolic compounds play a key role in the complex interactions between fungi and plants, acting as both defensive agents and signalling molecules (Ferruz et al. 2016; Kulik et al. 2017).

### Nitrogen and mycotoxin biosynthesis

Soil nutrient and substrate composition can influence the chemical diversity of the mycotoxins produced. In soils, growth of plants and microorganisms is limited by inorganic N namely ammonium (NH_4_^+^) and nitrate (NO_3_^−^). Plant secondary metabolites, polyphenolic compounds, are known to shape the N-metabolism in soils, being a strategy by the plants as a chemical weapon to ensure habitat dominance and prevent other species from colonizing the niche (Girardi et al. 2022). Hättenschwiler and Vitousek (2000) described polyphenols as an evolutionary response to environmental stress since they are well known regulators of soil processes such as nitrification, as well as decomposition and nutrient recycling and control the pool and form of nutrients available for plants and/or microbes. In this context, the role of mycotoxin in the demand of N-species has not been elucidated. However, evidence shows that mycotoxin production is linked to nutrient sources.

Different authors have shown that N-fertilization significantly affected the production of DON *in planta* i.e. Arata et al. (2022), Beyer et al. (2006); Lemmens et al. (2004). They suggest that the mycotoxin levels are dependent on the chemistry of the N-source as well as on plant growth stage and yield. In soils, N is a valuable resource with soil microbiome being part of the N-cycle and N-metabolism. The processes of mineralization, the conversion of organic N (i.e. amino acids, aminosugars) into NH_4_^+^, followed by nitrification, the oxidation of NH_4_^+^ into NO_3_^−^ via NO_2_^−^, are key steps in energy sources and biomass development for plants and microorganisms. Ammonium (NH_4_^+^) is directly assimilated by microorganisms and energetically more favorable than NO_3_^−^ or organic N, since the assimilatory reduction/mineralization to NH_4_^+^ requires energy (Geisseler et al. 2010). Mycotoxigenic fungi can also be part of N-metabolism in soil, with the potential to convert N-sources and simultaneously producing mycotoxins.

In particular for *Fusarium spp*., the level of DON produced can be influenced by the N-source: NO_3_^−^ by acting as a repressor of *TRI5* induction can favour DON production (Gardiner et al. 2009). Competitive dynamics of *Fusarium* species (i.e. *F. solani*, *F. sambucinum*, and *F. moniliforme*) result in the use of both forms of inorganic N (Celar 2003). The author assumed that *Fusarium spp.* are better adapted to various N-environments, compared to other species that utilize NO_3_^−^ only when NH_4_^+^ is depleted. In this context, testing for mycotoxins in these processes is imperative. A decrease in *F. oxysporum *f. sp*. dianthi* population in Baarn and ‘s-Gravenzande soil after supply with 0.1% or 1% NH_4_Cl or 0.1% urea was observed compared to the controls. The authors draw a relation to the nitrification in the respective soils (Löffler et al. 1986), suggesting an ecological role of DON in the dynamics of NO_3_^−^ and NH_4_^+^.

Similar evidence is shared for other mycotoxins: The production of FB1 by *F. fujikuroi* was stimulated under N-depletion (Shim and Woloshuk 1999). This was confirmed by Kohut et al. (2009) and Kim and Woloshuk (2008), who also investigated the genes involved in scenarios of N-depletion. *F. oxysporum* growth on plate was reduced under low supplementation of inorganic nitrogen compounds (≤ 50 mM), yet accompanied by autophagy events that enhanced mycotoxin-related metabolism (Karpe et al. 2018). But not only depletion, but also N-metabolism goes along with the production of mycotoxins. Nitrification by aflatoxigenic strains was significantly higher (122.7 ± 9.3 mg NO_3_-N L^− 1^) than that produced by non-toxigenic strains (50.8 ± 16.0 mg L^− 1^ N-NO_3_^−^) (White and Johnson 1982). This result indirectly suggests the involvement of AFB1 in nitrification (Shih et al. 1974) similar to secondary plant metabolites. In an incubation experiment, Wang et al. (2017) showed that organic N-sources i.e. glutamine are more effective in producing AFB1 compared to N-mineral sources. This may indicate that the fungi (*A. flavus*) increase the levels of mycotoxin towards more efficient N sources via mineralization. Other similar case: inorganic N inhibited AOH and AME synthesis by *Alternaria alternata* compared to nitrogen supplied as the amino acids phenylalanine (Brzonkalik et al. 2011). Tenuazonic acid production was not affected by C: N rations but AOH production showed an optimum production at a C: N of 72 (Brzonkalik et al. 2012).

In agricultural fields, a competition for N between plants and microorganisms exists, but has been only indirectly investigated in relation to mycotoxins. During plant development of strawberries, higher levels of the mycotoxin NIV were observed in the 10–30 cm soil –coinciding with the root zone– compared to topsoil (Meyer et al. 2020, 2022). Root growth was indicated by increased exudation of organic carbon, as plants allocate a significant fraction of the assimilated C to the soil for nutrient uptake, microbial attractions of beneficial microbes and defence (Kumar et al. 2006). Stresses such as nitrogen depletion or temperature changes may activate oxidative stress pathways and induce DON production in *Fusarium* species in soil (Ponts et al. 2006).

Mycotoxins are compounds which mediate chemical signalling within the microbial consortium (Venkatesh and Keller 2019). Therefore, mycotoxins should be further investigated for their ecological relevance in biogeochemical processes, which depend upon nitrogen sources and availability. Especially, the ecological role of toxins and the chemical factors that trigger their biosynthesis need to be in the focus of subsequent research, considering that these toxins are the most frequently detected mycotoxins in soils.

### Fungicides trigger the production of mycotoxins

Fungicides have been a constant technology in agriculture for decades, whose use is frequently justified because of economic improvements. However, fungicides act directly against microorganisms, by eliciting an adaptive ecological response (Riedo et al. 2023). Responses are also observed with filamentous fungi, showing enough evidence that the type of fungicide and application rate can reduce fungal biomass but not necessarily the biosynthesis of mycotoxins.

The first studies on an impact of fungicide concentrations on the chemotype of mycotoxins produced were conducted in the late 1990s. *F. culmorum* insensitive species were found to be more active in the production of 3-ADON than sensitive species upon exposure to difenoconazole (D’Mello et al. 1997, 1998). Similar results were reported later by Wegulo et al. (2011) when *F. graminearum* was exposed to the active substances prothioconazole and tebuconazole (formulated as Prosaro^®^ 421 SC). On the other hand, a reduction of DON concentrations was achieved when the same fungicide formulation was applied at anthesis to manage Fusarium head blight (FHB) (Willyerd et al. 2012). These findings indicate a developmental stage-dependent effectivity of these fungicides in reducing fungal infection and DON accumulation. Triazole fungicides, a class within the demethylation inhibitors (DMI) group, trigger oxidative stress in *F. graminearum* (Audenaert et al. 2010), which in turn trigger the biosynthesis of Fusarium toxins (Ponts 2015). After 48 h of prothioconazole incubation at sublethal doses, an increase in DON production was found in both, plate assays and plant experiments (Audenaert et al. 2010). Myclobutanil, reduced *F. verticilloides* mycelium growth, but along with an accumulation of DON after exposure (racemic mixture) at EC_50_ concentrations (Li et al. 2018). An stereoselective production of DON by *F. graminearum* was observed with the SS and SR isomers of cyproconazole (He et al. 2021), duplicating the production of DON compared to the racemic mixture. Similar results were reported for the fungicide flutriafol (Li et al. 2021), with DON accumulation shaped by temperature and water activity. Cyproconazole and myclobutanil are no longer approved in Germany for plant protection.

Similar to triazole, the effectiveness of strobilurins is doubtful, due to an increase of mycotoxins linked to it (de Chaves et al. 2022). Azoxystrobin, a quinone outside inhibitor (QoI) fungicide, reduced the colonization by toxigenic *Fusarium* species but increased DON levels (Paul et al. 2018; Simpson et al. 2001). In soils, a significant increase in frequency and concentration of DON was observed after fungicide application in strawberry cultivation, and attributed to fungicide (fenhexamid, fludioxonil and cyprodinil) induced stress (Meyer et al. 2022) as the highest levels of DON and 15-ADON coincided with the highest fungicide load (fludioxonil and cyprodinil) along with a significant reduction in fungal biomass (Meyer et al. 2021).

Given that fungicides affect soil microbial activity and that additional pest management practices can influence their kinetics and persistence (Ghosh and Singh 2009), it is important to assess how fungicide concentrations in soil correlate with peaks in the mycotoxin production *in situ*, particularly in soils with high population densities of *Fusarium sp.* Besides the efficiency of fungicides against *Fusarium* diseases in crops, strains resistant to agricultural fungicides can contribute to increased mycotoxin production (de Chaves et al. 2022). This should not only be investigated at plant levels, but also at soil level considering that *Fusarium* is part of the soil microbiome and fungicides when applied to crops may reach the soil and trigger a mycotoxin production *in situ*.

### Plant secondary metabolism impact the biosynthesis of mycotoxin *in planta*

Studies investigating phenolic acids, including cinnamic acid, aim to elucidate their role in altering mycotoxin biosynthesis in Fusarium-infected maize. For instance, Ferrigo et al. (2021) found that phenolic compounds like ellagic acid downregulate the expression of genes involved in fumonisin production, indicating a direct influence on the *FUM* gene biosynthetic pathway. Similarly, Martínez-Fraca et al. (2022) identified an association between phenolic acid content in maize pericarp and resistance to Fusarium ear rot, particularly noting that ferulic acid correlates with reduced fumonisin accumulation. Research by Ferruz et al. (2016) expanded on these findings by demonstrating that several phenolic acids, including ferulic and p-coumaric acids, inhibit mycotoxin production and fungal growth across various *Fusarium* species. Meanwhile, Kulik et al. (2017) explored the conversion and degradation of exogenous phenolic acids like trans-cinnamic and chlorogenic acids by *Fusarium* species, revealing complex interactions that impact on their antifungal efficacy. Additionally, studies aimed at understanding mechanistic details, emphasize the critical role of phenolic acids in regulating certain genes. The downregulation of mycotoxin biosynthesis-related genes, such as those in the *TRI* and *FUM* gene clusters, has been evidenced in response to phenolic acid treatment, suggesting a molecular basis for observed reductions in mycotoxin levels (Atanasova-Penichon et al. 2014; Boutigny et al. 2010; Ponts et al. 2011). Overall, the current body of research provides insights into the potential of employing phenolic acids as natural inhibitors of mycotoxin production, with implications for developing resistant maize varieties and integrating natural fungicidal treatments into crop management strategies. Further exploration into the specific molecular pathways and environmental influences on phenolic acid efficacy remains necessary to refine these approaches and maximize their utility in agricultural practices.

Hormones effect the interplay between pathogenic fungi and host plants as well (Chanclud and Morel 2016). Both the fungi and the plant are capable to produce hormones. In plants, salicylic acid (SA), jasmonic acid (JA) and ethylene (ETH) pathways are known to be involved in plant defence responses against pathogens e.g. (Bari and Jones 2009; Broekaert et al. 2006; Glazebrook 2005). However, less is known about the roles of the plant derived hormones cytokinins (CK), gibberellins (GAs), or auxins (IAAs) (Iqbal et al. 2024). Chanclud and Morel (2016) investigated the various potential roles of hormone production in pathogenic fungi. They propose that fungal derived hormones may operate through two potential mechanisms: The hormones may influence plant processes to facilitate invasion and nutrient uptake. Additionally, they may serve as signals for the fungi, triggering appropriate developmental and physiological responses suited to their environment.

*Fusarium* species have been shown to be capable of synthesizing GAs, CKs and auxins and might participate in pathogenicity (Vrabka et al. 2019). However, comparative genomics of strains of the *F. fujikuroi* species complex (FFC) that were shown to infect maize seedlings, revealed the presence of gene clusters capable for the production of GAs (Niehaus et al. 2016). Tsavkelova et al. (2012) provided evidence that *Fusarium sp.* are involved in fungal auxin production. An infection with *Fusarium* induced the expression of host plant cytokinin biosynthesis genes in maize seedlings. In parallel, *CKX* genes were expressed to reduce the endogenous cytokinin level that was considered as a protective mechanism against the parasite (Vrabka et al. 2019). The induction of cytokinin synthesis by pathogenic fungi plays a crucial role in regulating source–sink relationships in the host plant (McIntyre et al. 2021), auxin and cytokinins could therefore be regulators required for virulence. *F. pseudograminearum* was demonstrated to produce four specific hormones named fusatin, 8-oxo-fusatin, fusatinic acid and 8-oxo-isopentenyladenine, which were assigned to a new subclass of cytokinins. All four were shown to activate cytokinin signaling *in planta* and reprogram host development (Sørensen et al. 2018). In *F. proliferatum*, two homologs of the GA cluster were found (Niehaus et al. 2016) and GAs were isolated and identified from a culture filtrate of *F. fujikuroi* (Hedden and Sponsel 2015). Vrabka et al. (2019) examined the accumulation of these hormones *in planta* during a *Fusarium*–host plant interaction, their metabolism and their possible contribution to disease symptoms. It turned out that some disease symptoms are clearly linked to fungal hormone production.

*Fusarium* mycotoxins have also been implicated in plant pathogenicity. *Fusarium* species produce fusaric acid, which inhibits bacterial quorum sensing by disrupting acyl-homoserine lactone signalling. Additionally, DON has been identified as a virulence factor in plant interactions with various *Fusarium* species (Venkatesh and Keller 2019). It is known, that mycotoxins, such as DON and its derivatives, play a significant role in pathogenicity during wheat infection (Kotowicz et al. 2014). However, their role in corn colonization remains less clear. When maize seeds were exposed to AFB1 and AFG1, endogenous levels of GA-like substances, *trans*-zeatin (t-Z) and IAA decreased in the germinating seeds (Ağar et al. 2006). GA at a concentration of 2 ppm was found to be more effective in stimulating the length of root and shoot in AFB1-treated maize seeds than in none treated ones (Prasad et al. 2022). Sphingolipids have been recognized as one of the major membrane lipid components in eukaryotic cells (Sperling and Heinz 2003). Furthermore, sphingolipids participate in plant developmental regulation, stimulus sensing, and stress responses. FB1 was shown to disturb the plant sphingolipid metabolism (Liu et al. 2021) and imitating programmed cell death. Inhibition of ceramide synthesis by FB1 causes mislocalization of auxin carrier proteins by inducing small vesicular structures like dismantled vacuoles or fused vesicles in plant cells (Markham et al. 2011). On the other hand, Qin et al. (2017) then reported that Phyto-sphingosine (phyto-sph) activated JA and Phyto-sphingosine-1-phosphate (phyto-S1P) could initiate the SA signaling pathway, and together they altered plant resistance to FB1 stress. Further research should establish whether or not fungal-derived hormones act like other fungal effectors. There is also a need for further research in the field of plant and fungi derived hormones and their interaction with mycotoxins. Another need is the interplay of the plant response with abiotic factors such as nitrogen levels and pesticide strategies, as they are known to affect the physiology of the plant and of the pathogenic fungi e.g. Dieye et al. (2024).

## Future consideration for a risk evaluation of mycotoxins in environmental samples

### Suitable analytical methods and sampling strategies

The number of studies investigating mycotoxin levels in soils is limited to few studies, with reported concentrations about 100–1000 times lower than levels reported in plants or food commodities. Soil is a complex matrix whose physical, chemical and microbial heterogeneity depends on several factors, such as climate and geography (Soudzilovskaia et al. 2015), cover crop (Ding et al. 2006) or agricultural management (Haruna and Nkongolo 2019). Furthermore, micro-scale variations can be observed in the soil properties, which may affect the distribution of pollutants (Blanco-Canqui and Lal 2007, 2008). Suitable monitoring strategies, including sampling which consider the complexity of soil as matrix and patterns of mycotoxin distribution in soils are imperative. An additional factor to be considered when investigating mycotoxins in soils is the accessibility to suitable analytical methods, capable of differentiating between families of mycotoxins and metabolites and allowing reliable quantification of mycotoxins in different types of soils. Standardized analytical methods are needed to reduce uncertainty of data and facilitate a comparison between studies.

Only few studies have so far reported levels of mycotoxins in soils: Accinelli et al. (2008) and Hariprasad et al. (2015) reported AFB1 in soils at concentrations up to 20 µg kg^− 1^. Analysis was done using HPLC-Fluorescence and TLC combined with icELISA for quantitative analysis, respectively. But information on limits of detection and reproducibility of the method is unfortunately missing. Mortensen et al. (2003) developed and validated a method for an analysis of OTA and ZEN concentrations in soils (HPLC-Fluorescence), however these mycotoxins were not detected in real soil samples. The authors suggested a fast degradation of the mycotoxins in soils rather than considering a methodological error, as the method fulfilled the quality criterion. So far, DON and ZEN were successfully recovered from different soils using a mixture of acetonitrile/water/glacial acetic acid as extraction solution (Kappenberg and Juraschek 2021), and applied to environmental samples with values for DON and ZEN < 10 ng g^− 1^. Kenngott et al. (2022) showed that the soil type influences the extraction efficiency for ZEN but not for DON, NIV or 15-ADON using the generic combination of acetonitrile/water (84 + 16 v/v). LC-MS analysis showed that polar mycotoxins such as DON are more influenced by ions from the soil matrix than mycotoxins with larger retention times i.e. 15-ADON or ZEN. The authors also applied the method to estimate the occurrence of mycotoxins in maize cropped soils, observing a heterogeneous distribution of mycotoxins in the investigated soils. Albert et al. (2021) developed a simple method for the extraction of aflatoxins using the generic mixture acetonitrile/water (84 + 16 v/v), for analysis via HPLC-fluorescence and LC-HRMS. The method was demonstrated to be suitable for the analysis of different soils regardless of clay content and for an analysis of aflatoxins in some cereals. Previously, a method consisting of extraction with methanol and water and LC-HRMS analysis was validated and applied to asparagus (Muñoz et al. 2015) and strawberry (Meyer et al. 2021; Muñoz et al. 2017) cultivated soils.

The above mentioned studies focus on the detection of regulated mycotoxins and do not consider metabolites or transformation products i.e. hydrolysis, deacetylation, oxidation, conjugates, etc. Metabolites as those from soil biotransformation or plant metabolism should be included in order to avoid underestimation of environmental data. Monitoring of soils for mycotoxins and its key metabolites can be used as a putative biomarker of levels in crops as well as the efficiency of the terrestrial ecosystems to reduce environmental levels and for a robust prediction of a translocation from soil to plant.

### Mycotoxin degradation in soils facing scenarios of climate change and intensive agriculture

Microbial degradation is the most effective way of mycotoxin dissipation in soils, but the efficiency of the process may be reduced with increasing concentrations. Climate change is suggested to worsen the contamination scenario regarding mycotoxins in different geographic regions. Perrone et al. (2020) forecasted a shift of microbiome structure and dominance with a change in the geographical distribution of fungi and an increase in the production of mycotoxins. Moretti et al. (2019) proposed three major scenarios for mycotoxins in Europe under climate change: (1) contamination of maize with aflatoxins will be a major issue; (2) DON contamination in wheat as an emerging problem in Northern Europe; (3) *Fusarium* species causing FHB of wheat undergo continuous changes in fungi and mycotoxin profiles. The main source of mycotoxins entering aquatic and terrestrial ecosystems is the run-off from contaminated crops, as described previously by Juraschek et al. (2022). The aforementioned scenarios under climate change will result in a greater number of mycotoxins and higher concentrations reaching the soil. The first step to consider is whether a degradation or transformation of mycotoxins occurs, and under which conditions, since soil processes and functions are key for the half-lives of mycotoxins in soils. There is sufficient evidence demonstrating the impact of climate change on microbiome structure, C and N pools and soil functions with regard to degradation e.g. Jansson and Hofmockel (2020), Hamidov et al. (2018). The drainage of mycotoxin-contaminated runoff water through the soil column may represent a natural and effective pathway to reduce mycotoxin levels, when mycotoxins are accessible for microbial degradation or mineralization. In such cases, mycotoxins can be efficiently degraded by soil microorganisms, using them as carbon source under carbon limitation or nutrient stress. However, efficiency of using mycotoxins as a carbon source depend on microbial groups as well as on soil activity.

Along with climate change, intensive agriculture may constrain soil processes by altering environmental conditions through the use of agrochemicals. In the last decades, soils have been subjected to increasing stress to meet the increasing demands for food and feed production (Kopittke et al. 2019). An intensification of agriculture leads to an ongoing soil degradation, which limits the long-term ability of soils to provide essential ecosystem services to humans (Reganold et al. 1990). These services depend on the integrity of soil functions, and their value is often underappreciated (Jónsson et al. 2017). Therefore, our appeal is to include mycotoxin monitoring in soil and crops. In particular, at post-harvest when residues are left in the soil, this results in an opportunity for mycotoxigenic species to overwinter in the soil and to produce mycotoxins either in the soil or in the plant residues e.g. Juraschek et al. (2022). Considering the potential of soil to reduce levels of mycotoxins via biodegradation, the absence of mycotoxins in agricultural settings (i.e. cereal crops) can be considered as proxy for soil quality and functioning of processes.

### Interplay between secondary metabolites from fungi and plant

Secondary metabolites from fungi can provide an advantage of the producing fungi compared to other competing species. Mycotoxins are often in some cases complex chemical structures whose biosynthesis is linked to an energy requirement for the producing species. Therefore, their synthesis is not spontaneous and should theoretically be linked to some functionality. We have observed that mycotoxigenic fungi respond for nutrient deficiencies such as N by mycotoxin production. Considering that a producing fungus spends a large part of its life cycle in the soil, these secondary metabolites must be interpreted in a more evolutionary way (Venkatesh and Keller 2019). One of soil processes relevant for plant and microorganism is the processes of nitrification. Mycotoxins, as secondary metabolites of fungi, may exert effects analogous to the those of plants with allelopathic properties (Lotina-Hennsen et al. 2006). Further, there is enough evidence suggesting that *Fusarium* growth (Orr et al. 2022) as well as mycotoxin production are related to fertilization management. We suggest to further investigate the potential of mycotoxins from *Fusarium* in processes such as mineralization, nitrification and assimilation to better understand the ecological function of mycotoxins in soils. As well as to elucidate the reasons why an important fraction of the fungal biomass mobilizes to plant reaching pathogenic levels, probably in nutrient exhaustion in the soil.

Secondary metabolites from plants have also an ecological function in terrestrial systems. They are also involved in the response to *Fusarium* in plants. Innovative crop systems with inter-cropping or crop rotation have been suggested to reduce *Fusarium* severity as well as mycotoxin incidence in plants i.e. as observed by Drakopoulos et al. (2021) with white mustard or Indian mustard as intercrops reducing DON in winter wheat by up to 52%. *Desmodium* intercropping in push–pull farming was effective in managing maize ear rots and FBs in maize crops in western Kenya (Njeru et al. 2020). Mechanisms associated to those responses are the presence of plant secondary metabolites. Phenolic compounds obtained from botanical extracts have a key function in combating fungi and mycotoxins as reviewed by Ahmed et al. (2022). For example, synergistic effects of catechin, kaempferol, epicatechin, gallic acid, and quercetin were demonstrated for their potential to inhibit FB1 production (Vinas et al. 2025). Treatment of soils with olive mill wastewater did not reduce the fungal growth but AFB1 production (Bavaro et al. 2016). Waste water from olive oil production is rich in polyphenolic compounds (Carrara et al. 2021) and is used as accepted agricultural management. Other soil managements rich in polyphenolic compounds are grape pomace (Buchmann et al. 2025) or residues from tea cropping (Tsubaki et al. 2008). Considering that polyphenolic compounds modulate N- processes such as nitrification (Girardi et al. 2022) or denitrification (Bardon et al. 2017), this chemical advantage of the polyphenolic compounds can be used to modulate the *Fusarium* response and the mycotoxin production towards a reduction of synthetic pesticides in crops.

## Conclusions

In soils, mycotoxin levels represent only a minimal fraction of the initial input from contaminated materials. Once in the soil, mycotoxins are exposed to abiotic and biotic factors in several processes: adsorption to soil particles, (bio)degradation, transport/mobilisation and uptake by plants. Mycotoxins mobilise with water from contaminated crops, regardless of the polarity of the molecule. Upon entering soil, adsorption to clay or organic matter occurs through non-covalent interactions, resulting in partial immobilisation of the mycotoxins and reduced availability for soil (bio)degradation. For molecules with carbonyl functions, adsorption to clay is more relevant than that to organic matter. Mycotoxins with a negative Log*K*_ow_, i.e. DON/NIV show a reduced adsorption to soil. There is a body of evidence, that the main removal pathway for mycotoxins is microbial degradation and mineralisation, a process that is universal for several mycotoxins such as DON, NIV, 15-ADON, AFB1, ZEN, and OTA. Therefore, it is reasonable to assume that the quality of soils and the integrity of the soil microbiome are critical in achieving temporary low levels of mycotoxins. Plant uptake has been confirmed for several mycotoxins in incubation experiments without soil. However, there is still limited information available to draw conclusions for realistic field scenarios. *In planta* and in soils, mycotoxigenic fungi are exposed to stress scenarios such as nutrient limitation or pesticides, which can lead to a transient increased of mycotoxin biosynthesis. The plant response involves also metabolism of the mycotoxin and the synthesis of secondary metabolites i.e. polyphenolic compounds. These observations underline the importance of safeguarding microbial diversity and soil function, not only for agricultural productivity, but also for maintaining the resilience of biogeochemical cycles in the face of global change. We conclude that meeting this challenge requires an integrated approach starting in the soil, combining soil health monitoring, microbial biodiversity conservation, and sustainable land management, such as regenerative farming, supportive policies and ongoing research in this area.

## Data Availability

No datasets were generated or analysed during the current study.
